# Surfactant-free Synthesis of CuO with Controllable Morphologies and Enhanced Photocatalytic Property

**DOI:** 10.1186/s11671-016-1278-z

**Published:** 2016-03-03

**Authors:** Xing Wang, Jiao Yang, Liuxue Shi, Meizhen Gao

**Affiliations:** Key Laboratory for Magnetism and Magnetic Materials of MOE, School of Physical Science and Technology, Lanzhou University, 730000 Lanzhou, People’s Republic of China

**Keywords:** Copper oxide, Ammonia, Morphology, Photocatalytic activity

## Abstract

A green synthesis for nanoleave, nanosheet, spindle-like, rugby-like, dandelion-like and flower-like CuO nanostructures (from 2D to 3D) is successfully achieved through simply hydrothermal synthetic method without the assistance of surfactant. The morphology of CuO nanostructures can be easily tailored by adjusting the amount of ammonia and the source of copper. By designing a time varying experiment, it is verified that the flower- and dandelion-like CuO structures are synthesized by the self-assembly and Ostwald ripening mechanism. Structural and morphological evolutions are investigated by X-ray diffraction (XRD), scanning electron microscopy (SEM) and UV-visible diffuse reflectance spectra. Additionally, the CuO nanostructures with different morphologies could serve as a potential photocatalyst on the photodecomposition of rhodamine B (RhB) aqueous solutions in the presence of H_2_O_2_ under visible light irradiation.

## Background

The photocatalytic performance, electrical and gas-sensing properties are strongly influenced by their morphology and size. Many investigations have been carried out to study the controlling of size, morphology and structure of materials during synthesis [1–4]. To achieve this, the studies of the crystal growth, morphology evolution processes and the corresponding mechanisms are significantly important. As an important p-type transition-metal oxide with a narrow band gap varying between 1.2 and 1.8 eV [5], CuO has been widely studied in thermal conductivity [6], optoelectronic device systems [7], CO oxidation [8], eradication of multi-drug resistant bacteria [9], Li ion batteries anodes [10], heterogeneous catalyst for olefin epoxidation [11], gas sensing [12, 13] and glucose sensor [14–18]. In the past decade, CuO with different morphologies such as nanoribbons [12], microworms [13], nanoplatelets [19], dandelions [20], sandwich [16], nanowires [17], nanotube arrays [21], nanourchins [2, 18] and nanorods [22] have been successfully synthesized through different methods with the assistance of surfactant such as CTAB, PVP, PEG and SDS. Since the surfactants invariably present residual surfactants or organic additives attached to the surfaces of products can block the active sites, it is a serious issue when considering applications in gas sensing or catalysis. Therefore, it is still a challenge to develop new green surfactant-free methods to synthesize well-defined CuO nanostructures [23]. Zhang et al. [24] synthesized flower-like CuO microspheres by a hydrothermal route at 130 °C for 18 h without the assistance of surfactant. Sun et al. [23] synthesized two-dimensional CuO mesoplates and three-dimensinonal CuO mesospindles by an additive-free complex-precursor solution route.

Inspired by the green methods to synthesize various controllable CuO morphologies with surfactant-free building blocks, we present a simply low temperature hydrothermal synthetic method without the assistance of surfactant. And spindle-like, rugby-like, nanoleaves, nanosheets, microspheres and dandelions CuO nanostructures are synthesized through this green method. The synthesis is performed in an ethanol-water mixed solvent using copper source and ammonia as the variable. By designing a time varying experiment, it is verified that the flower- and dandelion-like CuO structures are synthesized by the self-assembly and Ostwald ripening mechanism. X-ray diffraction (XRD), scanning electron microscope (SEM) and UV-visible diffuse reflectance spectra are employed to characterize the obtained CuO nanostructures. Furthermore, these copper oxide nanostructures are found to be high qualified photocatalysts for the degradation of rhodamine B (RhB) under visible light irradiation in the presence of hydroxide water (H_2_O_2_).

## Methods

### Materials and synthesis

Cu(NO_3_)_2_ · 3H_2_O (Cheng Du Kelong Chemical Reagent Company), Cu(COOH)_2_ · H_2_O (Tianjin Guangfu Fine Chemical Research Institute), NH_3_ · H_2_O (25 wt% ~ 28 wt%) (Cheng Du Kelong Chemical Reagent Company) and rhodamin B (RhB) (Tianjin Guangfu Fine Chemical Research Institute) are analytical grade and used without further purification. For a typical CuO nanostructure synthesis, 0.604 g (0.25 mol) of Cu(NO_3_)_2_ · 3H_2_O or 0.5 g (0.25 mol) Cu(COOH)_2_ · H_2_O mixed solution of 60 mL of deionized water and alcohol (the ratio of alcohol and water is 1:1) and stirred until completely dissolved. At the same time, an appropriate amount of NH_3_ · H_2_O is added into the above solution quickly and stirred 30 min. The resulting mixture is then transferred into a Teflon-lined steel autoclave and heated in an oven at 80 °C for 10 h. Finally, the precipitates are separated by centrifugation, washed with deionized water and alcohol for several times and dried at 70 °C for 12 h. Through this method, different morphology and size CuO is synthesized by adjusting the amount of NH_3_ · H_2_O and copper source. The variable parameters are listed in Table 1.

**Table 1:** The morphologies and synthesis parameters of CuO nanostructures

Sample no.	The amount of NH_3_ · H_2_O (mL)	Copper source	Morphologies
A	0.5	Cu(NO_3_)_2_ · 3H_2_O	Spindle-like
B	0.5	Cu(COOH)_2_ · H_2_O	Rugby-like
C	0.6	Cu(NO_3_)_2_ · 3H_2_O	Nanoleave
D	0.6	Cu(COOH)_2_ · H_2_O	Nanosheet
E	1.5	Cu(NO_3_)_2_ · 3H_2_O	Dandelion-like
F	1.5	Cu(COOH)_2_ · H_2_O	Flower-like

### Characterization

Powder XRD pattern is recorded on an X’Pert Philips diffractometer (Cu Kα radiation: λ = 1.5418 Å, 2θ range 20∼80°, accelerating voltage 40 kV, applied current 150 mA). The morphology of the products is investigated by field emission scanning electron microscopy (SEM, Hitachi S-4800).

### Photocatalytic properties

The photocatalytic activity of the CuO nanostructures with different morphologies is evaluated by the degradation of a model pollutant RhB under the visible light irradiation with the assistance of hydrogen peroxide (H_2_O_2_) at ambient temperature. The original solution is prepared by mixing 5 mL H_2_O_2_ (30 wt%), 50 mL RhB solution (10^−5^ M) and 20 mg copper oxide powder together and then stirred in the dark for 60 min to ensure an adsorption-desorption equilibrium is established. Afterwards, the dispersion is irradiated by a 350-W xenon lamp equipped with a filter cutoff (λ ≥420 nm) under magnetic stirring. At given time intervals, the dispersion is sampled and centrifuged to separate the catalyst. The adsorption spectrum of the solution is then recorded with an UV-visible spectrophotometer (Shimadzu UV-3600).

## Results and discussion

### Crystal structures of the prepared CuO nanomaterials

**Fig. 1:**
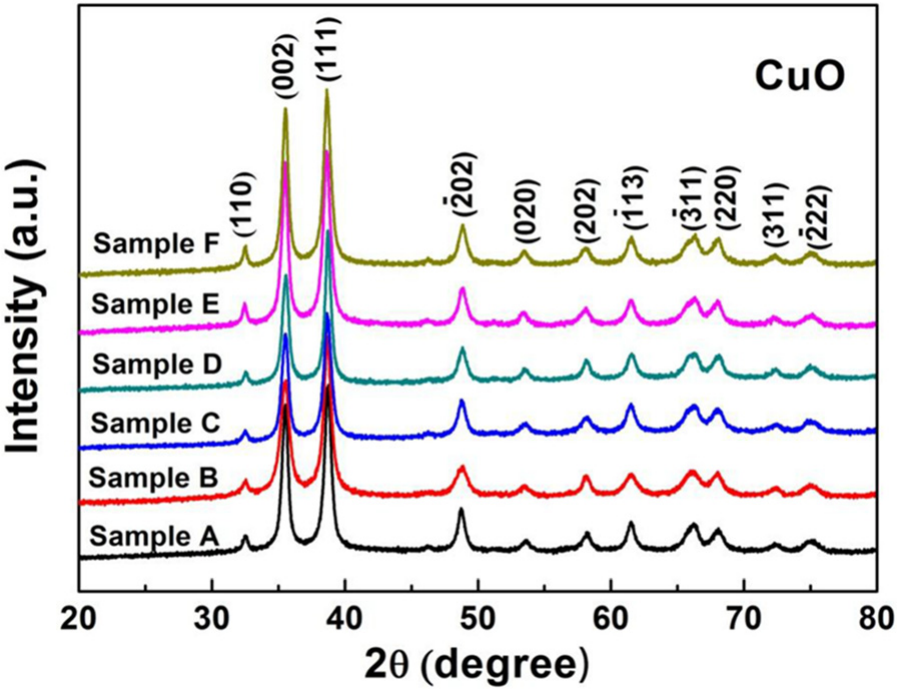
XRD patterns of CuO nanostructures after hydrothermal treatment with different reaction conditions

Figure 1 shows the XRD patterns of the products under different reaction conditions after hydrothermal treatment. Diffraction peaks are observed at 2θ of 32.5°, 35.5°, 38.7°, 48.7°, 53.7°, 58.2°, 61.5°, 66.3°, 68°, 72.3° and 74.8°. These peaks can be assigned to the (110), (002), (111), (-202), (020), (-113), (-311), (220), (311) and (-222) planes of monoclinic CuO (JCPDS 65-2309). No peaks of impurities such as copper hydroxide or other copper compounds can be detected, suggesting the high purity and similarity of all the as-prepared products.

### Morphologies of CuO nanostructures synthesized by Cu(NO_3_)_2_ as a copper source

**Fig. 2:**
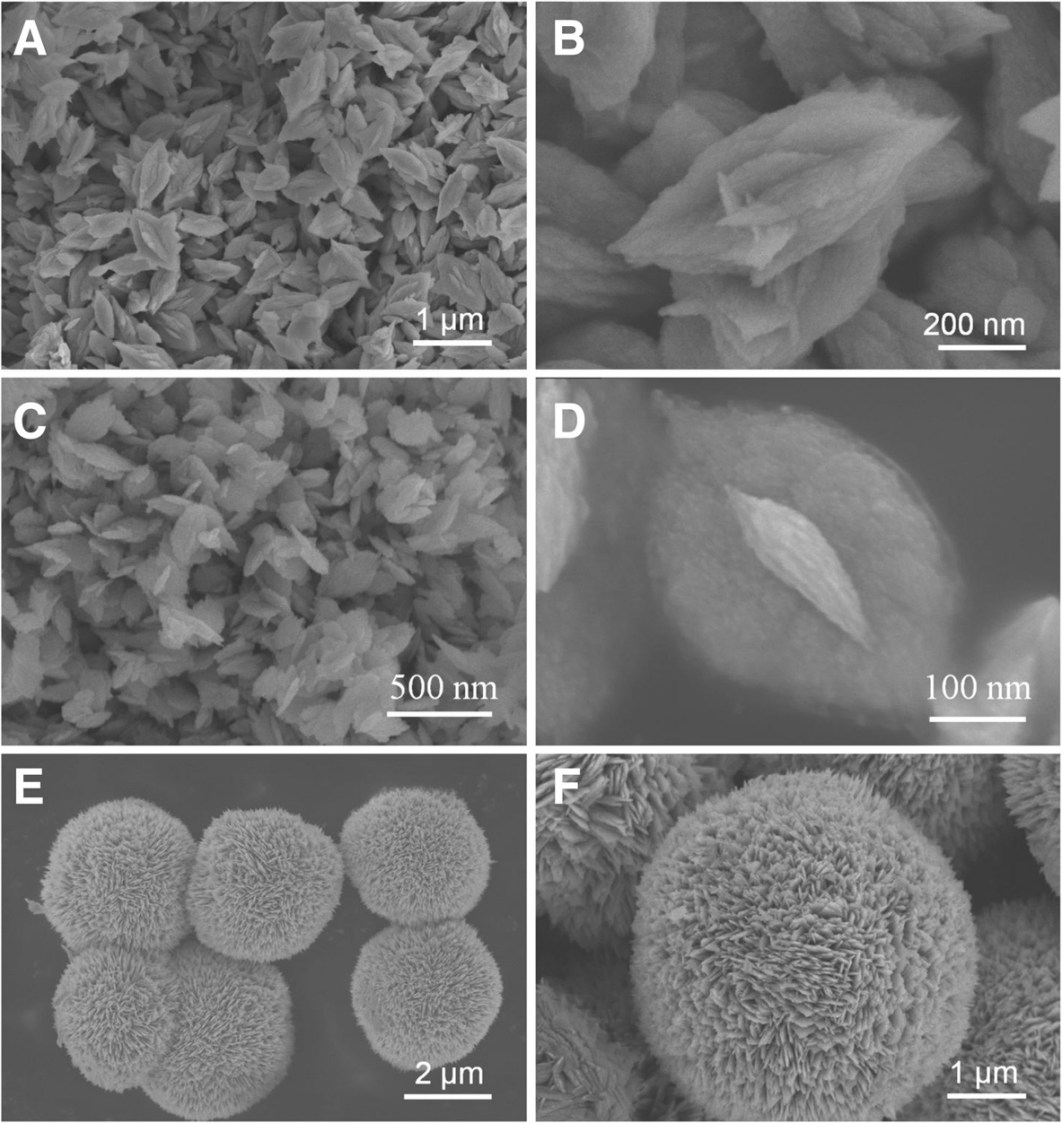
Electron microscopy images of CuO obtained by different amount of ammonia: **a**, **b** sample A; **c**, **d** sample C; **e**, **f** sample E

When 0.5 mL of NH_3_ · H_2_O is added in the reaction system, CuO nanostructure with a spindle-like morphology is produced as shown in Fig. 2a and Fig. 2b. We can see that the CuO nanostructures are about 600 nm in length and 300 nm in width. When the volume of the NH_3_ · H_2_O is increased to 0.6 mL, the centre of the spindle-like CuO is getting thinner and finally obtains a large number of nanoleaves (Fig. 2c, d). The CuO nanostructures are about 250 nm in length and 130 nm in width. Further increase NH_3_ · H_2_O to 1.5 mL, 3D dandelion-like CuO with hierarchical nanostructures is obtained (Fig. 2e, f). The diameter of the CuO is about 4 μm. As can be seen from the SEM, the dandelion-like CuO is consisted of nanosheets (Fig. 2f). But further increases in the amount of NH_3_ · H_2_O, the morphology and size of the CuO do not change significantly, all the products remain dandelion-like.

### Morphologies of CuO nanostructures synthesized by Cu(COOH)_2_ as a copper source

**Fig. 3:**
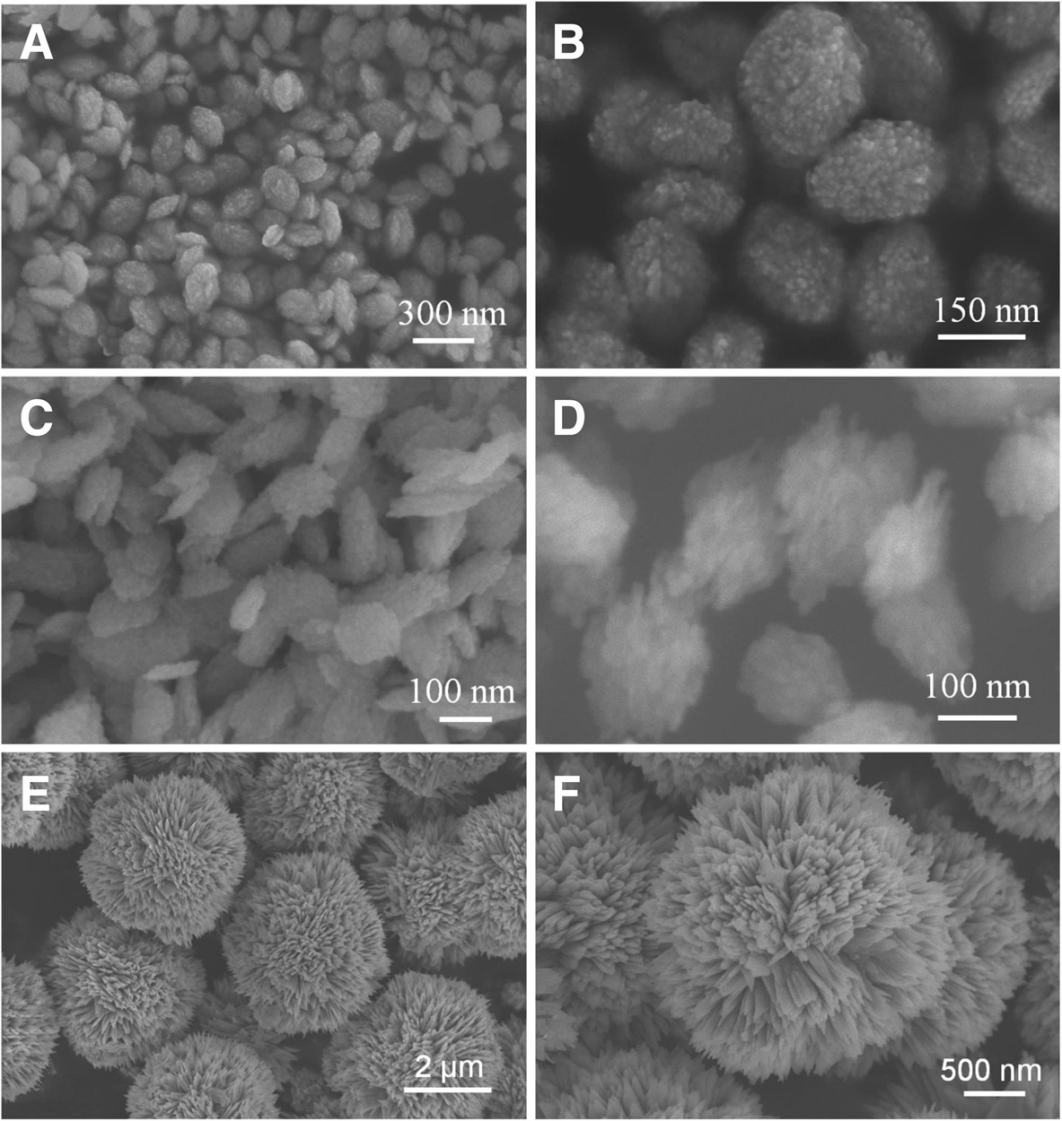
SEM images of CuO obtained by different volume of ammonia: **a**, **b** sample B; **c**, **d** sample D; **e**, **f** sample F

When 0.5 mL of NH_3_ · H_2_O is added in the reaction system, CuO nanostructures with rugby-like morphology are produced as shown in Fig. 3a and Fig. 3b. The CuO nanostructures are about 200 nm in length and 130 nm in width. When the volume of the NH_3_ · H_2_O is increased to 0.6 mL, a large number of nanosheets with about 150 nm in length and 100 nm in width can be obtained (Fig. 3c, d). When the amount of the NH_3_ · H_2_O is increased to 1.5 mL, 3D flower-like CuO microstructures consisted of nanosheets with the diameter about 3 μm can be obtained (Fig. 3e, f). The accumulation of nanosheets of dandelion-like CuO is denser than the flower-like CuO, and the diameter of dandelion-like CuO is larger than that of flower-like CuO. Further increase the amount of NH_3_ · H_2_O, the morphology and size of the CuO do not change anymore, all the products remain flower-like CuO microstructures.

### Plausible mechanisms for the formation of CuO nanostructures

When 0.5 mL or 0.6 mL ammonia is added in to the solution, a flocculent precipitation is generated in these blue solutions. The precursor in the blue solution before heat treatment is Cu(OH)_2_. Copper oxide is obtained after heat treats the Cu(OH)_2_ precipitation; the reactions can be summarized as follows:

1 $$ \mathrm{C}{\mathrm{u}}^{2+} + 2\mathrm{N}{\mathrm{H}}_3\cdotp {\mathrm{H}}_2\mathrm{O}\to \mathrm{C}\mathrm{u}{\left(\mathrm{O}\mathrm{H}\right)}_2\downarrow + 2\mathrm{N}{{\mathrm{H}}_4}^{+} $$

2 $$ \mathrm{C}\mathrm{u}{\left(\mathrm{O}\mathrm{H}\right)}_2\ \overset{80{}^{\circ}\mathrm{C}}{\to}\mathrm{C}\mathrm{u}\mathrm{O} + {\mathrm{H}}_2\mathrm{O} $$

Increase the dose of ammonia further, the firstly formed flocculent precipitate dissolved. Then [Cu(NH_3_)_4_]^2+^ can be considered as the precursor entity for the formation of CuO. The reactions can be described as follows:

3 $$ \mathrm{C}{\mathrm{u}}^{2+} + 4\mathrm{N}{\mathrm{H}}_3\cdotp {\mathrm{H}}_2\mathrm{O}\ \to\ {\left[\mathrm{C}\mathrm{u}{\left(\mathrm{N}{\mathrm{H}}_3\right)}_4\right]}^{2+} + 2\mathrm{O}{\mathrm{H}}^{-} + 4{\mathrm{H}}_2\mathrm{O} $$

4 $$ {\left[\mathrm{C}\mathrm{u}{\left(\mathrm{N}{\mathrm{H}}_3\right)}_4\right]}^{2+} + 2\mathrm{O}{\mathrm{H}}^{-}\overset{80{}^{\circ}\mathrm{C}}{\to }\ \mathrm{C}\mathrm{u}\mathrm{O} + 4\mathrm{N}{\mathrm{H}}_3 + {\mathrm{H}}_2\mathrm{O} $$

In the present case, CuO particles are synthesized directly by the decomposition of Cu(OH)_2_ or (Cu(NH_3_)_4_)^2+^ precursor under hydrothermal conditions without the presence of various surfactant. The crystal formation process can be divided into two stages: nucleation and crystal growth. When the amount of ammonia is less than 0.6 mL, Cu(OH)_2_ is formed in aqueous reaction medium, which transformed into CuO under hydrothermal conditions [Eqs. (1) and (2)]. When the amount of ammonia is over 1.5 mL, the soluble [Cu(NH_3_)_4_]^2+^ complex is formed, which transformed into CuO under hydrothermal conditions [Eqs. (3) and (4)]. The different growth unite might affect the competition between thermodynamics and kinetics during the reduction of precursors and nucleation and growth of CuO crystals [25]. Comparing Fig. 2 with Fig. 3, we can conclude that the morphologies of CuO are not the same and the sizes of CuO become smaller when the copper source changes from Cu(NO_3_)_2_ to Cu(COOH)_2_. The effect of copper source on the structure of CuO may be that the NO_3_^−^ is inorganic strong acid root and the COOH^−^ is organic weak acid root; in the synthesis of complex precipitation, the anions in the solution affect the nucleation and growth of the copper oxide precursor. From the above analysis, it is safe to say that the ammonia and the acid radical ion have an important effect on the formation of CuO morphology.

**Fig. 4:**
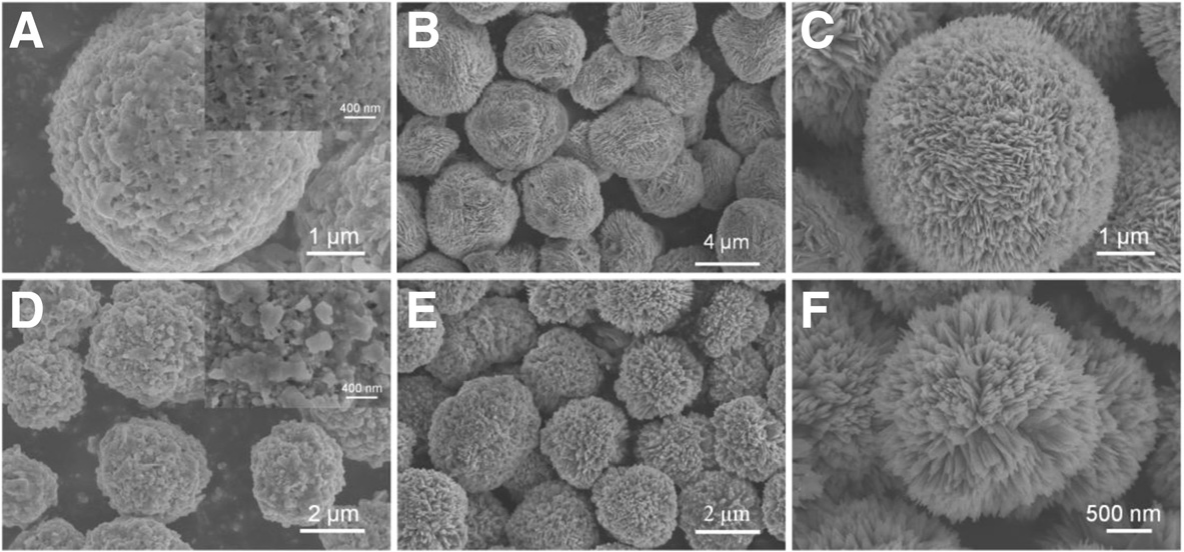
SEM images of CuO obtained after different reaction time: **a**, **d** 3 h; **b**, **e** 5 h; **c**, **f** 10 h

Both self-assembly and Ostwald ripening mechanism are involved in the process of synthesizing of the dandelion-like and flower-like CuO. To verify this, the time-dependant morphologies of the dandelion- and flower-like CuO are shown in Fig. 4. We can conclude that after 3 h, CuO microspheres are formed by the self-assembly of particles, but the surface of microspheres is uneven; there are still many small particles attached to the surface of microspheres (Fig. 4a, d). With the extension of reaction time, these particles gradually ripe and the final structures are formed (Fig. 4c, f).

### Photochemical performances

**Fig. 5:**
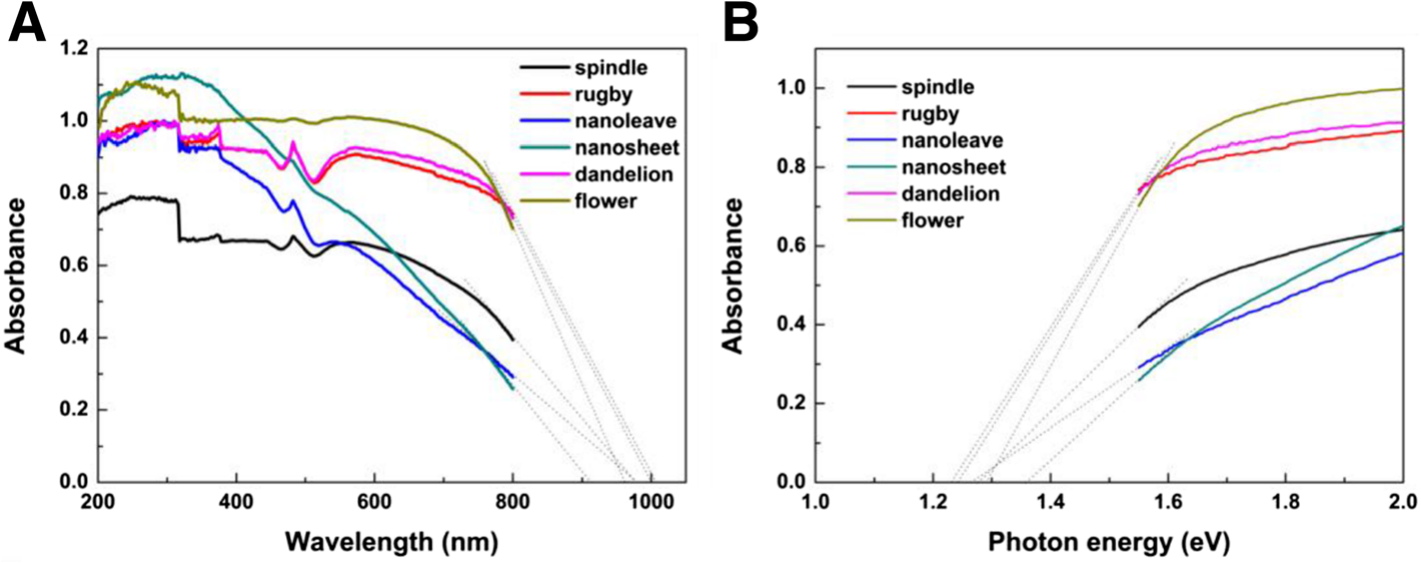
**a** UV-vis spectra and (**b**) plot of photon energy of as-prepared CuO products with different morphologies

The optical energy band gaps of semiconductors are found to be dependent on their microstructures, so the above as-prepared CuO nanostructures with different morphologies are investigated by UV-vis spectra. Figure 5a shows the UV-visible spectra of as-prepared CuO products with different morphologies, the absorption edges of spindle-like, rugby-like, nanoleave, nanosheet, dandelion-like and flower-like CuO occur at 975, 1010, 985, 910, 1000 and 960 nm, respectively. Figure 5b shows the plot of photon energy; we can find that the band gaps of spindle-like, rugby-like, nanoleave, nanosheet, dandelion-like and flower-like CuO are estimated to be 1.27, 1.23, 1.26, 1.36, 1.24 and 1.29 eV, respectively. The effect of the morphology of CuO nanostructure is clearly noted on its band gap. The fundamental photodegradation mechanism involves the acceleration in decomposition of H_2_O_2_ over CuO crystals to generate free radical species, such as .OH, .OOH or .O_2_^−^, which are deemed to be liable for the degradation of the dyes. The related chemical reactions include the electrons (e^−^) in the VB can be excited to the CB and at the same time generate the same number of holes (h^+^) in the VB. The formed e^−^ and h^+^ pairs can be captured by H_2_O_2_ molecules leading to the formation of .OH, .HOO or .O_2_^−^ [Eqs. (5)–(8)]; oxidant species react with dye then finally realize complete mineralization with the formation of CO_2_, H_2_O or other inorganic ion [23].

5 $$ \mathrm{C}\mathrm{u}\mathrm{O} + \mathrm{h}\upnu \to {\mathrm{h}}_{\mathrm{vb}+} + {\mathrm{e}}_{\mathrm{c}{\mathrm{b}}^{-}} $$

6 $$ {\mathrm{H}}_2{\mathrm{O}}_2 + {\mathrm{h}}_{\mathrm{vb}+}\to {\kern0.5em }^{.}\mathrm{O}\mathrm{O}\mathrm{H} + {\mathrm{H}}^{+} $$

7 $$ {\mathrm{H}}_2{\mathrm{O}}_2 + {\mathrm{e}}_{\mathrm{c}{\mathrm{b}}^{-}}\to {\kern0.5em }^{.}\mathrm{O}\mathrm{H} + \mathrm{O}{\mathrm{H}}^{-} $$

8 $$ {}^{.}\mathrm{O}\mathrm{O}\mathrm{H}\to {\kern0.5em }^{.}{{\mathrm{O}}_2}^{-} + {\mathrm{H}}^{+} $$

**Fig. 6:**
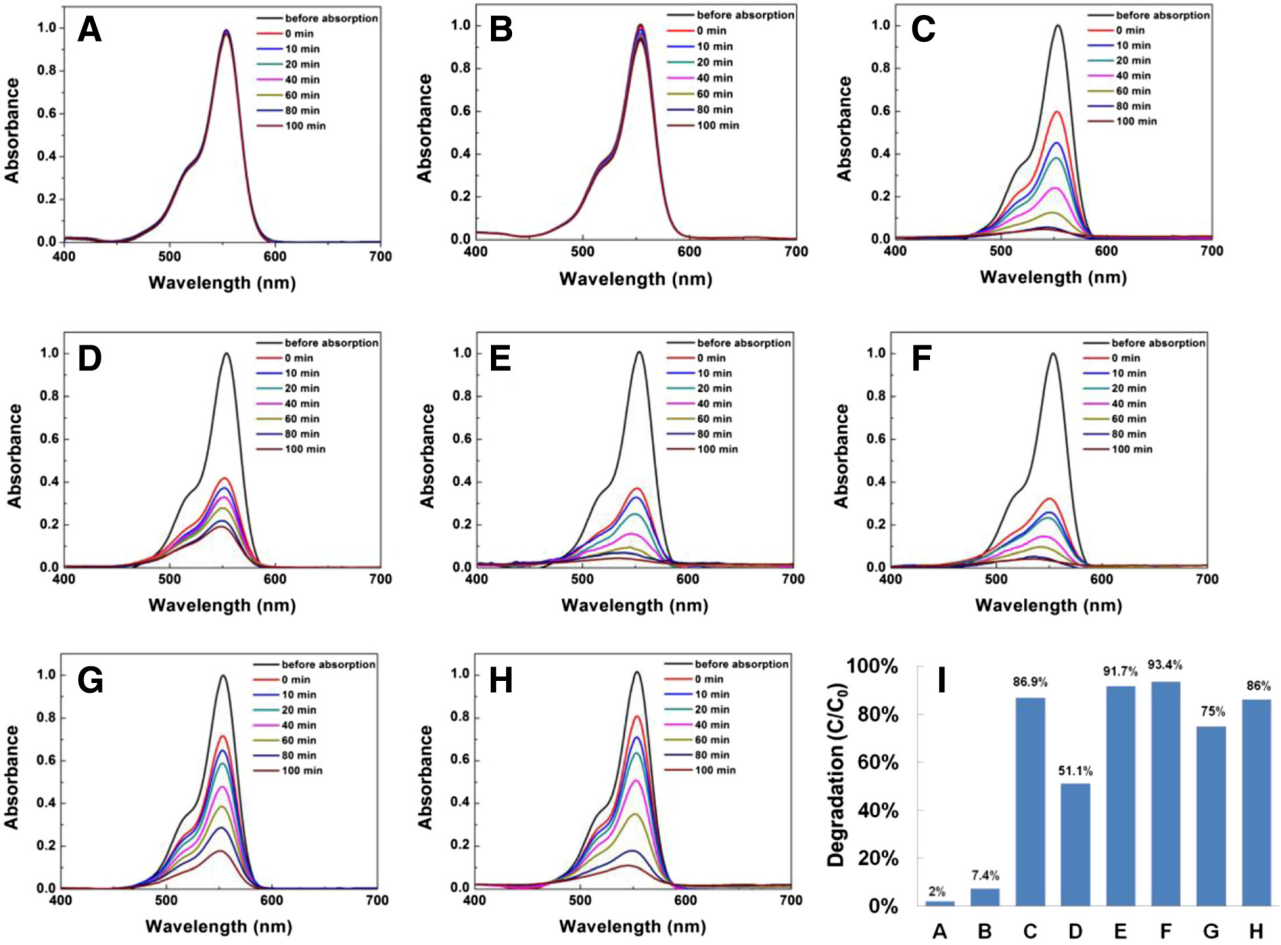
Absorption spectra of RhB under visible light irradiation with different CuO nanostructures. **a** Without any catalyst and H_2_O_2_, **b** only with H_2_O_2_, **c** with sample A, **d** with sample B, **e** with sample C, **f** with sample D, **g** with sample E, **h** with sample F, and **i** plot of the extent of photodegradation of RhB which corresponds to *A*, *B*, *C*, *D*, *E*, *F*, *G* and *H*

To explore the photochemical performances of the as-prepared CuO nanostructures, photocatalytic activity of CuO is evaluated in the oxidation of RhB under visible light irradiation at ambient temperature in the presence of hydroxide water (H_2_O_2_). The characteristic absorption pick at 554 nm of RhB is monitored to follow the catalytic degradation process. Figure 6a shows the optical absorption spectra of RhB measured at different intervals in the absence of catalysts and H_2_O_2_; only a slight degradation (2.2 %) of RhB can be detected after 100 min (Fig. 6i, *A*). Figure 6b shows the optical absorption spectra of RhB measured at different intervals only with the H_2_O_2_; only 7.4 % of RhB can be detected after 100 min (Fig. 6i, *B*). Figure 6c–h shows the optical absorption spectra of RhB tested at different intervals in the presence of different nanostructured CuO. It can be found that the degradation rate of nanoleave and nanosheet CuO are both very fast, 91.7 and 93.4 % are reached respectively after 100 min (Fig.6i, *E*, *F*). The lowest degradation rate is about 51.1 % by the rugby-like CuO (Fig. 6i, *D*). The degradation of RhB at 100 min in the presence of above six samples is as follows: nanosheet CuO (93.4 %) > nanoleave CuO (91.7 %) > spindle-like CuO (86.9 %) > flower-like CuO (86 %) > dandelion-like (75 %) > rugby-like (51.1 %).

## Conclusions

In summary, a green synthesis for spindle-like, rugby-like, nanoleave, nanosheet, dandelion-like and flower-like CuO nanostructures (from 2D to 3D) are successfully achieved through simply hydrothermal synthetic method without the assistance of surfactant. The formation of CuO nanostructures here is basically effected by the amount of ammonia and the copper source. We also found that the flower- and dandelion-like CuO structures are synthesized by the self-assembly and the Ostwald ripening mechanism. Additionally, the CuO nanostructures with different morphologies could serve as a potential photocatalyst on the photodecomposition of RhB aqueous solutions in the presence of H_2_O_2_ under visible light irradiation.
